# Stress Management Apps: Systematic Search and Multidimensional Assessment of Quality and Characteristics

**DOI:** 10.2196/42415

**Published:** 2023-08-29

**Authors:** Sarah Paganini, Evelyn Meier, Yannik Terhorst, Ramona Wurst, Vivien Hohberg, Dana Schultchen, Jana Strahler, Max Wursthorn, Harald Baumeister, Eva-Maria Messner

**Affiliations:** 1 Department of Sport Psychology Institute of Sports and Sport Science University of Freiburg Freiburg Germany; 2 University of Education Freiburg Freiburg Germany; 3 Clinical Psychology and Psychotherapy Ulm University Ulm Germany; 4 Department of Sport, Exercise and Health Faculty of Medicine University of Basel Basel Switzerland; 5 Clinical and Health Psychology Ulm University Ulm Germany; 6 Department of Public and Nonprofit Management University of Freiburg Freiburg Germany

**Keywords:** stress management, mobile app, mHealth, mobile health, quality assessment, review, evidence base, availability

## Abstract

**Background:**

Chronic stress poses risks for physical and mental well-being. Stress management interventions have been shown to be effective, and stress management apps (SMAs) might help to transfer strategies into everyday life.

**Objective:**

This review aims to provide a comprehensive overview of the quality and characteristics of SMAs to give potential users or health professionals a guideline when searching for SMAs in common app stores.

**Methods:**

SMAs were identified with a systematic search in the European Google Play Store and Apple App Store. SMAs were screened and checked according to the inclusion criteria. General characteristics and quality were assessed by 2 independent raters using the German Mobile Application Rating Scale (MARS-G). The MARS-G assesses quality (range 1 to 5) on the following four dimensions: (1) engagement, (2) functionality, (3) esthetics, and (4) information. In addition, the theory-based stress management strategies, evidence base, long-term availability, and common characteristics of the 5 top-rated SMAs were assessed and derived.

**Results:**

Of 2044 identified apps, 121 SMAs were included. Frequently implemented strategies (also in the 5 top-rated SMAs) were psychoeducation, breathing, and mindfulness, as well as the use of monitoring and reminder functions. Of the 121 SMAs, 111 (91.7%) provided a privacy policy, but only 44 (36.4%) required an active confirmation of informed consent. Data sharing with third parties was disclosed in only 14.0% (17/121) of the SMAs. The average quality of the included apps was above the cutoff score of 3.5 (mean 3.59, SD 0.50). The MARS-G dimensions yielded values above this cutoff score (functionality: mean 4.14, SD 0.47; esthetics: mean 3.76, SD 0.73) and below this score (information: mean 3.42, SD 0.46; engagement: mean 3.05, SD 0.78). Most theory-based stress management strategies were regenerative stress management strategies. The evidence base for 9.1% (11/121) of the SMAs could be identified, indicating significant group differences in several variables (eg, stress or depressive symptoms) in favor of SMAs. Moreover, 38.0% (46/121) of the SMAs were no longer available after a 2-year period.

**Conclusions:**

The moderate information quality, scarce evidence base, constraints in data privacy and security features, and high volatility of SMAs pose challenges for users, health professionals, and researchers. However, owing to the scalability of SMAs and the few but promising results regarding their effectiveness, they have a high potential to reach and help a broad audience. For a holistic stress management approach, SMAs could benefit from a broader repertoire of strategies, such as more instrumental and mental stress management strategies. The common characteristics of SMAs with top-rated quality can be used as guidance for potential users and health professionals, but owing to the high volatility of SMAs, enhanced evaluation frameworks are needed.

## Introduction

Stress is a public health problem that poses high risks for physical and mental well-being and is increasing in industrial societies where individuals are exposed to complex demands at work and in daily life [1-5]. The results of an American survey revealed that 75% of the participants felt significantly stressed [6], and a representative sample of the German population showed a point prevalence of perceived high chronic stress of 11% [1]. There are multiple reasons for the broad impact of stress as it affects many dimensions, such as cognition (eg, negative attributional style), affect (eg, affective dysregulation, such as increase in anxiety), physiology (eg, dysregulation of the endocrine response system), and behavior (eg, harmful behavioral changes, such as smoking or physical inactivity) [3]. As a result, chronic stress causes a higher risk for various somatic diseases and mental disorders, such as gastric ulcers, migraine, hypertension, type 2 diabetes mellitus, and depression [3,5,7-11]. In addition to the substantial impact on health, work-related stress results in high costs for society, especially through productivity-related losses [12].

Due to these negative consequences, several stress management strategies have been developed and evaluated over the past decades [13,14]. Most of them refer to transactional stress models in which stress reactions are mainly determined by a subjective interpretation and the types of coping strategies employed [13-16]. Even though “stress management” is a widely and variably used term [17], Kaluza [13] proposed three categories of strategies: (1) instrumental stress management strategies with a focus on preventing and reducing stress in everyday life (eg, self-management or seeking support); (2) mental stress management strategies aiming at changing personal stress amplifiers (eg, acceptance or gratitude); and (3) regenerative stress management strategies aiming at recovery after stress exposure (eg, relaxation techniques or health behavior) [17-19]. Thereby, effective stress management seems to be characterized by a broad repertoire and a balance between instrumental, mental, and regenerative strategies [15]. Interventions use a variety of these different strategies and are often delivered in a group setting [17,18]. In particular, cognitive or behavioral-based interventions for stress management (which typically include all 3 categories of strategies) have been shown to be effective for reducing stress in different settings (eg, the occupational setting; Cohen *d*=1.16, 95% CI 0.46-1.87 [20]) and for different target groups (eg, university students; standardized mean difference=−0.77, 95% CI −0.97 to −0.57 [21]). The same is true for mindfulness-based stress reduction in healthy individuals [22-24] (eg, Hedges *g*=0.53, 95% CI 0.41-0.64 [22]). Relaxation training (with a focus on regenerative strategies) has been shown to be effective in healthy individuals [24] and in occupational settings (Cohen *d*=0.50, 95% CI 0.31-0.69 [20]) but appears to be inferior to cognitive-behavioral interventions [20]. Implementing previously learned health-related strategies in daily life is essential for their short- and long-term health benefits [25]. Internet- and mobile-based health interventions can help to integrate stress management strategies into daily routines and to overcome the barriers of face-to-face interventions, such as limited accessibility, location, time, and high costs [26,27]. As a result, the relevance of mobile phones for monitoring and delivering health interventions has increased over the last decade [27]. From 2013 to 2018, the number of downloaded health apps per year increased from 1.7 to 4.1 billion worldwide [28]. Regarding stress management apps (SMAs), about 6% of an American sample reported that they already use SMAs regularly and about 50% could imagine using them in the future [29]. An observational study showed that compared to a website, delivering a stress-management intervention via an app could offer the added benefit of more frequent use and access to more intervention content [30].

Accordingly, there is a broad and growing body of SMA research. Reviews already exist; however, they all focus on specific aspects and some might be outdated. Pre-existing SMA reviews focus on content alone [31,32], content in combination with transparency and functionality [33], efficacy [34], gamification elements [35], persuasive and behavior change strategies [36], or quality of apps, with a focus exclusively on mindfulness apps [37]. Regarding content, it was shown that mindfulness and meditation were the most commonly used strategies in the reviewed SMAs (34% to 78% of all apps included these strategies) [31,33,34], followed by breathing [31,33] or goal setting [34]. Further common strategies were personalization and self-monitoring, while social support strategies were rarely used [36]. The implementation of gamification elements is relatively scarce (on average 0.5 elements per app) [35]. Concerning the evidence base, Lau et al [34] revealed that among more than 1000 screened apps for well-being and stress management, only 2% were scientifically evaluated. The 2 studies that looked at data privacy and security features, such as privacy policy, contact information, and disclosures, revealed that only half of these criteria could be met on average [33]. In addition, most of the evaluated apps showed a lack of data privacy and security [37]. This confirms the results of other health, wellness, and medicine-related apps (eg, smoking cessation and diabetes) [38-40], showing major privacy and security risks, missing transparency, or data sharing with third parties, even when they were accredited [41].

Considering the quality of SMAs, only one of the existing reviews (which was exclusively performed for mindfulness apps and not for SMAs in general) [37] employed a valid scientific measure, that is, the Mobile Application Rating Scale (MARS), which is an instrument for assessing app quality on multiple dimensions [42,43]. This is of relevance as user star ratings and app store descriptions can be manipulated in favor of commercial interests [33].

Another challenge for users and health professionals who are seeking apps for long-term use, as well as for researchers aiming to present the most recent state of research, is the high volatility of apps [44,45]. In a study considering mental health apps, only 50% of the search results were available at the end of a 9-month period [44]. Considering the excessive supply of health apps in app stores, their high update rate, and their uncertain long-term availability, as well as the current lack of transparency of app quality [46], the question arises as to which SMAs should be used and recommended.

In light of all these gaps and issues, the aim of this study was to systematically search for SMAs, to assess their quality on multiple dimensions in a scientific manner, and to give a comprehensive overview of SMAs concerning their general characteristics, theory-based stress management strategies, evidence base, and long-term availability. A further aim was to inform potential users or health professionals about common characteristics that might indicate high quality of SMAs. The following research questions were addressed:

1. What are the general characteristics of SMAs, such as descriptive information, technical aspects, strategies, and functions?
2. What is the quality of SMAs regarding multiple dimensions (ie, engagement, functionality, esthetics, and information)?
3. Which theory-based stress management strategies are used in SMAs?
4. What is the evidence base of SMAs?
5. How reliable are SMAs in terms of their long-term availability?
6. What are the common characteristics of SMAs with top-rated quality?

## Methods

### Overview

This study involved a systematic search and assessment of the quality and characteristics of SMAs. It was registered in the Open Science Framework (OSF) of the Center for Open Science [47].

### Search Strategy and Procedure

The search terms were generated through a 3-step process. First, a narrative literature search was conducted to collect terms and keywords that were used in studies focusing on SMA interventions for the general population. Second, relevant search terms were identified based on interest group interviews with 3 psychotherapists and 3 potential SMA users. Third, the identified search terms from the literature search and results of the interest group interviews were merged, leading to the following search terms: “stress,” “stress management,” “stress reduction,” “stress prevention,” “stress coach,” “stress recovery,” “relaxation,” and “relaxation training.” An automated search using these search terms (in English and German language) was conducted in the European Apple App Store and Google Play Store with a search engine (web crawler). It was developed as part of the Mobile Health App Database Project [48], and it automatically extracts information, such as app name, description, and user rating, from the stores (for further details, please see [49]).

Apps from both app stores were identified and listed in a central database. Duplicates were automatically removed. In the first step and based on the description in the app stores, apps were screened for the following inclusion criteria: (1) the word “stress” was included in the title or in the app store description; (2) the app was developed for adults in the general population without mental or somatic disorders; (3) the focus of the app was primarily on stress management; (4) at least two different stress management strategies were applied with the aim of including apps that potentially take a holistic approach to stress management; (5) the app could be used without further equipment, devices, or programs; (6) the app was free of cost in the basic version; and (7) the app was provided in German or English language. In the second step, the app was downloaded and rechecked for criteria 1 to 7. Apps that did not work after the download were excluded.

### Data Collection of General Characteristics and Quality Assessment

The general characteristics and quality of each SMA were collected and rated between March and May 2020 by 2 independent raters (EM, SP, Hannah Besel, RW, or VH) with the German Mobile Application Rating Scale (MARS-G) [42]. All raters had a psychology or sports science degree and completed an online training, which included the following components: (1) background information on the development of the MARS-G; (2) description of the dimensions and items; (3) application instructions; and (4) an exercise example [50]. Subsequently, 3 SMAs were assessed per rater in order to compare and discuss the results and to ensure a common standard as well as high data quality. According to the standardized procedure, each rater had to test an app for at least 15 minutes. Data collection and quality assessment, including the actual average time spent per rating, have been fully documented.

### General Characteristics

General characteristics were mainly based on the classification section of the original MARS and MARS-G [42,43] and included (1) descriptive information on the app (ie, app name, URL, platform, user star rating, full version price, content-related app category, declared aims of the app, theoretical background, and certification); (2) technical aspects (eg, links to social media or type of support); (3) strategies (eg, relaxation or goal setting); and (4) functions (eg, feedback or reminder).

Owing to the high relevance of data privacy and security, the list of the MARS-G [42] has been supplemented with 7 additional features (ie, passive informed consent; complex passwords; anonymization or pseudonymization; creation of an access token; automatic display of the privacy policy; permanent availability of the privacy policy; and transparency regarding the right of withdrawal [51,52]). The privacy policy of each included SMA was reviewed against the listed features. It was assessed whether information was provided for each feature (“yes” or “no”), but not whether it was technically and legally realized and complied with by the respective providers.

### Quality Assessment

The multidimensional quality evaluation based on the MARS-G [42] comprised 4 subscales: user engagement (5 items: entertainment, interest, customization, interactivity, and target group), functionality (4 items: performance, ease of use, navigation, and gestural design), esthetics (3 items: layout, graphics, and visual appeal), and information quality (7 items: accuracy of app description, goals, quality of information, quantity of information, quality of visual information, credibility, and evidence base). For the interpretation, the cutoff score of 3.5 (indicating above-average quality) defined by Terhorst et al [53] was used.

Additionally, the subjective quality (4 items: expected use frequency within 1 year, willingness to pay for the app, willingness to recommend the app, and subjective star rating) and the perceived impact on the user (6 items: awareness, knowledge, attitudes, intention to change, help-seeking, and behavioral change) were assessed. All items were rated on a 5-point Likert scale (1=inadequate, 2=poor, 3=acceptable, 4=good, and 5=excellent). The MARS is a well-validated instrument [43,54]. The validation of the German version also yielded excellent internal consistency (ω=0.84, 95% CI 0.77-0.88) and high levels of interrater reliability (interclass correlation [ICC]=0.83, 95% CI 0.82-0.85 [42]).

### Theory-Based Stress Management Strategies

To depict the variety of existing stress management strategies in more detail, we developed a list of theory-based stress management strategies [13,31]. This list contains 23 instrumental, mental, and regenerative stress management strategies (see Textbox 1). Some of the included theory-based strategies (eg, breathing, hypnosis, and mindfulness) overlapped with the strategies covered in the MARS-G. The assessment of theory-based stress management strategies was performed for each SMA by 2 independent raters.

**List of theory-based stress management strategies (adapted from Kaluza [13] and Christmann et al [31]).**

**Instrumental stress management strategies**

- Enhancing professional competencies (eg, learning)
- Seeking support (eg, network)
- Developing social-communicative skills (eg, self-assertion)
- Self-management

**Mental stress management strategies**

- Accepting reality (also included in the German Mobile Application Rating Scale [MARS-G])
- Seeing difficulties as challenges (not as threats)
- Changing personal stress amplifiers
- Self-efficacy

**Regenerative stress management strategies**

- Acupressure
- Autogenic training (the MARS-G includes the category “relaxation,” which is differentiated in more detail here)
- Biofeedback
- Breathing (also included in the MARS-G)
- Euthymic methods
- Food or nutrition
- Guided imagination or visualization
- Hypnosis or self-hypnosis (also included in the MARS-G)
- Meditation or mindfulness (also included in the MARS-G)
- Music
- Muscle relaxation (the MARS-G includes the category “relaxation,” which is differentiated in more detail here)
- Physical stress relief techniques
- Self-massage
- Sounds
- Sport (also included in the MARS-G)

### Evidence Base of Included SMAs and Long-Term Availability

All included SMAs were unsystematically searched in a common web search engine for scholarly literature by applying the app name and screening the first pages of the results. Information on study design, app usage in weeks, sample and target groups, age, gender, measurement time points, measured variables, and main results were assessed from the studies found (with the exception of pilot studies).

In terms of long-term availability, all included SMAs were searched again in the app stores in August 2022. It was checked whether the app was still available (on the original platform), when the last update was made, and whether the basic version was still free of cost.

### Characteristics of the 5 Top-Rated SMAs

Owing to the multitude of information, a concise overview of the common characteristics of the 5 top-rated SMAs (based on the MARS-G overall quality score) has been provided. This overview contains information on quality ratings, technical aspects, strategies and functions (all derived from the MARS-G), theory-based stress management strategies, evidence base, and long-term availability.

### Data Analyses

To ensure consistency between raters, the ICC (2-way mixed) was calculated according to the report by Koo and Li [55]. An ICC below 0.50 is considered poor, 0.51 to 0.75 is moderate, 0.76 to 0.89 is good, and above 0.90 is excellent [56]. For all descriptive data (such as aims, background, and data security features), frequency and percentage were calculated. The mean score and standard deviation have been presented for each dimension of the MARS-G. All analyses were performed using IBM SPSS Statistics (Version 21; IBM Corp).

## Results

### Search Results

**Figure 1:**
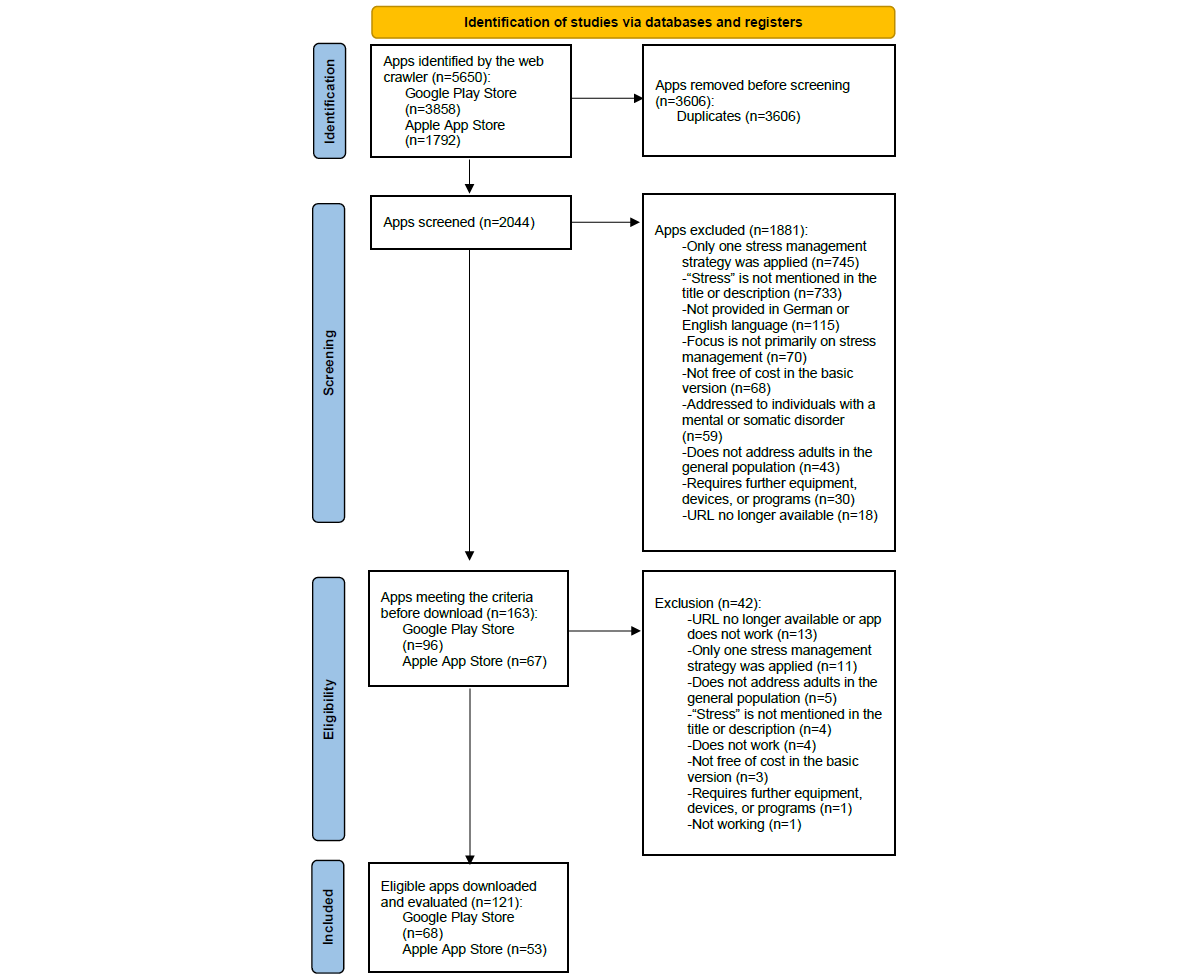
Flowchart according to the PRISMA (Preferred Reporting Items for Systematic Reviews and Meta-Analyses) statement 2020.

The web crawler identified 5650 potential SMAs (Google Play Store, n=3580; Apple App Store, n=1792). After removing duplicates, 2044 apps were screened. This screening resulted in 163 apps, of which 121 were eligible for inclusion after the download (Figure 1 [57]). On average, each SMA was used and evaluated for 30 minutes by each rater (mean 30.2, SD 4.0 minutes).

### General Characteristics and Quality Rating

#### Descriptive Information

Of the 121 included SMAs, 68 (56.2%) were derived from the Google Play Store and 53 (43.8%) from the Apple App Store. The user star rating in the app stores could be identified for 96 (79.3%) apps. The mean user star rating was 4.27 (SD 0.56), and the number of ratings per app ranged from 1 to 126,183. Of all rated SMAs, 83 (68.6%) could be upgraded to a premium version (from 1 month [with costs between 0.99 EUR and 15.99 EUR] up to a permanent upgrade [with costs between 1.09 EUR and 449.99 EUR]; 1 EUR=1.1197 USD). Regarding content-related categories, most apps were listed under “health and fitness” (107/121, 88.4%). Further assigned categories were “lifestyle” (6/121, 5.0%), “medicine” (6/121, 5.0%), and others (3/121, 2.5%; including “learning,” “entertainment,” and “audio/music”). According to the description, all apps aimed at reducing stress (121/121, 100%). Further aims were improvement of well-being (113/121, 93.4%), reduction of anxiety (90/121, 74.4%), improvement of physical health (50/121, 41.3%), and emotion regulation (58/121, 47.9%). Some SMAs focused on the reduction of depressive symptoms (33/121, 27.3%), behavioral change (21/121, 17.4%), and entertainment (3/121, 2.5%). Moreover, 81 (66.9%) apps reported additional goals, such as relaxation, increasing motivation and focus, improvement of sleep, and concentration or self-awareness. The most often assigned theoretical background was third-wave behavioral therapy (106/121, 87.6%), followed by behavioral therapy (30/121, 24.8%) and cognitive behavioral therapy (24/121, 19.8%). Overall, more than 100 SMAs (111/121, 91.7%) were developed with a commercial background, 5 (4.1%) were developed by a nongovernmental organization, and only few SMAs were developed by a university (2/121, 1.7%) or a governmental institution (1/121, 0.8%). No SMA was certified according to the European Union Medical Device Regulation [58].

#### Technical Aspects

Data exchange with other users (eg, via social media) was possible in 40 (33.1%) SMAs, and an app community existed in 20 (16.5%) SMAs. SMAs were unguided (74/121, 61.2%), technically guided (70/121, 57.9%), asynchronously guided by humans (4/121, 3.3%), or synchronously guided by humans (1/121, 0.8%). All data privacy and security features are presented in Table 1. The 3 most common features were provision of privacy policy (111/121, 91.7%), provision of contact details or imprint (111/121, 91.7%), and passive informed consent (80/121, 66.1%).

**Table 1:** Frequency of declared data privacy and security features based on the German Mobile Application Rating Scale [42].^a^

Data privacy and security feature	Number of apps that specify this feature (N=121), n (%)
Provision of a privacy policy	111 (91.7)
Contact or imprint	111 (91.7)
Passive informed consent^b^	80 (66.1)
Automatic display of the privacy policy^b^	76 (62.8)
Allows password protection	74 (61.1)
Requires login	66 (54.5)
Security of data transfer	64 (52.9)
Permanent availability of the privacy policy^b^	55 (45.5)
Active confirmation of informed consent	44 (36.4)
Financial background/conflict of interest	42 (34.7)
Transparency regarding the right of withdrawal^b^	34 (28.1)
Creation of an access token^b^	26 (21.5)
Complex passwords^b^	25 (20.7)
Data sharing with third parties	17 (14.0)
Security strategies in case of device loss	17 (14.0)
Emergency function	8 (6.6)
Anonymization or pseudonymization^b^	3 (2.5)
Place of storage	2 (1.7)

^a^A more descriptive presentation of the data can be found in Multimedia Appendix 1.

^b^Additional feature that has been added to the original list.

#### Strategies

As shown in Table 2, more than half of all 121 SMAs included the strategies breathing (95/121, 78.5%), relaxation (94/121, 77.7%), mindfulness or gratitude (91/121, 75.2%), information or education (83/121, 68.6%), and tips or advice (70/121, 57.9%).

**Table 2:** Frequency of the implemented strategies according to the German Mobile Application Rating Scale.^a^

Strategy	Number of apps that include the strategy (N=121), n (%)
Breathing	95 (78.5)
Relaxation	94 (77.7)
Mindfulness/gratitude	91 (75.2)
Information, education	83 (68.6)
Tips, advice	70 (57.9)
Acceptance	49 (40.5)
Physical exercises	38 (31.4)
Gamification	33 (27.3)
Skills, training	30 (24.8)
Resource orientation	24 (19.8)
Goal setting	22 (18.2)
Serious games	5 (4.1)
Hypnosis	4 (3.3)
Exposure	0 (0.0)

^a^A more descriptive presentation of the data can be found in Multimedia Appendix 2.

#### Functions

The most included function was monitoring or tracking (78/121, 64.5%), followed by reminder (76/121, 62.8%), data collection (49/121, 40.5%), feedback (48/121, 39.7%), and tailored intervention or real-time feedback (22/121, 18.2%).

#### Quality Rating

The agreement between raters was good (ICC=0.82, 95% CI 0.81-0.82). The mean overall quality score for the SMAs was 3.59 (SD 0.50; range 2.13-4.37), indicating acceptable to good quality exceeding the cutoff score of 3.5. The mean scores of the different dimensions were as follows: engagement, 3.05 (SD 0.78; range 1.40-4.60); functionality, 4.14 (SD 0.47; range 2.63-5.00); esthetics, 3.76 (SD 0.73; range 1.50-5.00); and information, 3.42 (SD 0.46; range 1.00-4.00). The mean score for subjective quality was 2.53 (SD 0.78; range 1.00-4.00) and that for perceived impact on the user was 2.64 (SD 0.77; range 1.25-4.25). The quality ratings of all SMAs can be found in Multimedia Appendix 3.

### Theory-Based Stress Management Strategies

The most common theory-based stress management strategies were meditation or mindfulness (80/121, 66.1%), breathing (61/121, 50.4%), music (31/121, 25.6%), guided imagination or visualization (26/121, 21.5%), accepting reality (25/121, 20.7%), and enhancing professional competencies (21/121, 17.4%). In the 121 SMAs, theory-based stress management strategies were included 355 times. The most implemented strategies were regenerative stress management strategies (on average, each strategy [n=15] was mentioned 17 times and implemented 248 times, ie, 70%), followed by mental stress management strategies (on average, each strategy [n=4] was mentioned 14 times and implemented 57 times, ie, 16%) and instrumental stress management strategies (on average, each strategy [n=4] was mentioned 13 times and implemented 50 times, ie, 14%). Table 3 shows the frequencies of the investigated theory-based stress management strategies.

**Table 3:** Theory-based stress management strategies according to Kaluza [13] and Christmann et al [31].^a^

Theory-based stress management strategy		Number of apps that include the strategy (N=121), n (%)
**Regenerative stress management strategy**		
	Meditation or mindfulness^b^	80 (66.1)
	Breathing^b^	61 (50.4)
	Music	31 (25.6)
	Guided imagination or visualization	26 (21.5)
	Sounds	20 (16.5)
	Food and nutrition	8 (6.6)
	Muscle relaxation	7 (5.8)
	Sport^b^	3 (2.5)
	Techniques for physical stress relief	3 (2.5)
	Autogenic training	3 (2.5)
	Self-massage	2 (1.7)
	Hypnosis or self-hypnosis^b^	2 (1.7)
	Euthymic methods	1 (0.8)
	Acupressure	1 (0.8)
	Biofeedback	0 (0.0)
**Mental stress management strategy**		
	Accepting reality^b^	25 (20.7)
	Seeing difficulties as challenges (not as threats)	17 (14.0)
	Self-efficacy	8 (6.6)
	Changing personal stress amplifiers	7 (5.8)
**Instrumental stress management strategy**		
	Enhancing professional competencies	21 (17.4)
	Self-management	18 (14.9)
	Developing social-communicative skills	6 (5.0)
	Seeking support	5 (4.1)

^a^A more descriptive presentation of the data can be found in Multimedia Appendix 4.

^b^Theory-based stress management strategy that is also included in the German Mobile Application Rating Scale.

### Evidence Base and Long-Term Availability

Scientific evaluations could be found in 11 (9.1%) of the 121 apps. Study designs varied and included randomized controlled trials (n=5), a partially randomized trial (n=1), a panel study (n=1), and pilot studies (n=3). One app was tested for its quality, and the results were summarized in a published conference paper (n=1). Target groups were university students (n=5), the general population (n=2), employed individuals (n=1), caregivers (n=1), adults with mild to moderate anxiety or depression (n=1), and nurses (n=1). Different health outcome variables were studied. In the 5 randomized controlled trials, significant group differences at postintervention in favor of the app could be found for the variables stress (n=2), self-efficacy (n=2), mindfulness (n=2), anxiety symptoms (n=4), and depression symptoms (n=4). Details of the evaluations (excluding the pilot studies and the conference paper) can be found in Multimedia Appendix 5 [59-77].

Two years after screening, 46 (38.0%) of the 121 SMAs were no longer available in the 2 app stores. Three apps did not exist anymore in English and were only available in another language (German). Nine apps were only available through other platforms that had less stringent review procedures compared with the official app stores. Among the 75 SMAs that were still accessible, 10 apps now had costs even in the basic version. Of the 121 SMAs, 46 (38.0%) had their last update in 2022 and 11 (9.1%) had not been updated since 2020.

### Characteristics of the 5 Top-Rated SMAs

The 5 apps with the highest overall MARS-G ratings are presented in Table 4. None of these apps was developed by a public institution (such as a government or university). However, in all apps, it was emphasized that they were developed by different experts (such as psychologists, psychotherapists, and neuroscientist), or researchers with experience in mindfulness, meditation, or coaching. Furthermore, 3 of these 5 SMAs were part of scientific studies (study design: randomized controlled trial). All apps integrated different forms of psychoeducation and information via text or audio, provided advice, and implemented breathing and mindfulness. Monitoring or tracking and reminders were also included in all apps. Additional theory-based stress management strategies were guided imagination or visualization and music. All apps were technically guided. Moreover, 3 of the 5 apps were tailored to the users’ needs based on a screening at the beginning or provided real-time feedback. Additionally, 1 SMA included messenger coaching and a contact list with therapists in different US states. Furthermore, 3 of the 5 apps offered an app community. All apps provided a specific login area (including password), privacy policy, and contact information or imprint. Moreover, 2 of the 5 apps offered an emergency function.

**Table 4:** Overview of the 5 top-rated apps.

Variable		App^a^				
		App 1	App 2^b^	App 3	App 4	App 5
Overall quality (MARS-G^c^)		4.36	4.22	4.21	4.19	4.16
**Quality dimensions (MARS-G)**						
	Engagement	4.30	4.10	3.80	4.00	4.30
	Functionality	4.63	4.38	3.50	4.75	4.25
	Esthetics	4.50	4.83	4.83	4.17	4.50
	Information	4.00	3.57	3.71	3.86	3.57
**Technical aspects (MARS-G)^d^**						
	Privacy and security features^e^	10	10	8	10	8
	Technical guidance	✓	✓	✓	✓	✓
	Tailored interventions, real time feedback	✓	✓		✓	
	App community	✓	✓			✓
**Strategies (MARS-G)^d^**						
	Information, education	✓	✓	✓	✓	✓
	Tips, advice	✓	✓	✓	✓	✓
	Breathing^f^	✓	✓	✓	✓	✓
	Mindfulness, gratitude^f^	✓	✓	✓	✓	✓
	Relaxation^f^		✓	✓	✓	
	Acceptance^f^	✓		✓		✓
**Functions (MARS-G)^d^**						
	Monitoring, tracking	✓	✓	✓	✓	✓
	Reminder	✓	✓	✓	✓	✓
	Data collection	✓	✓		✓	
	Automated feedback	✓	✓		✓	✓
**Theory-based stress management strategies^g^**						
	Guided imagination, visualization (RSMS^h^)	✓				✓
	Music (RSMS)		✓			✓
Evidence base^i^		✓	✓	✓		
**Long-term availability**						
	App still available after 2 years^j^	✓	✓	✓	✓	✓
Year of the last update		2022	2022	2022	2020	2022

^a^App 1, Happify: bei Ärger und Stress (English translation: Happify: Anger and Stress); App 2, Sanvello: Stress & Anxiety Help; App 3, Headspace: Meditation & Schlaf (English translation: Headspace: Meditation & Sleep); App 4, go4health – gesund leben (English translation: go4health – living healthy); App 5, BamBu: Meditation & Achtsamkeit (English translation: BamBu: Meditation & Mindfulness).

^b^Name of this app after the second search in August 2022: “Sanvello: Anxiety and Depression.” The app may no longer meet inclusion criterion 2 (“the app was developed for adults in the general population without mental or somatic disorders”). At the time of the screening process in 2020, we listed this app as “Sanvello: Stress & Anxiety Help,” and it met the inclusion criteria.

^c^MARS-G: German Mobile Application Rating Scale.

^d^With the exception of “privacy and security features,” all general characteristics of the categories “technical aspects,” “strategies,” and “functions” of the MARS-G are listed, which were included in at least three of the top 5 apps.

^e^The number of statements made regarding 19 possible security features is provided.

^f^Strategies that were included in the MARS-G and also in the list of theory-based stress management strategies.

^g^All theory-based stress management strategies (according to Kaluza [13] and Christmann et al [31]) are listed, when they were included in at least two of the top 5 apps. Theory-based stress management strategies that are already listed in the MARS-G strategies are not listed again (breathing [regenerative stress management strategy], relaxation [regenerative stress management strategy], mindfulness [regenerative stress management strategy], and acceptance [mental stress management strategy]).

^h^RSMS: regenerative stress management strategy.

^i^All studies were randomized controlled trials.

^j^App 4 was still available but only in the Google Play Store and was not available anymore in the Apple App Store, where it was found in 2020.

## Discussion

### Principal Findings

This systematic app search and standardized multidimensional assessment aimed to evaluate the general characteristics, quality, theory-based stress management strategies, evidence base, and long-term availability of SMAs. Furthermore, characteristics that might indicate high quality were derived from the 5 top-rated SMAs.

### General Characteristics

Learning and maintaining stress management strategies requires regular engagement for not only changing the stress-enhancing cognitions and emotions, but also changing behavior [14]. Most of the included SMAs support this learning process by providing information on the background of the intervention and thus about stress management, tracking progress, or the use of reminder functions. Especially, the frequent presence of reminder functions was found to be similar in previous reviews [78]. A subgroup analysis of apps for mental health problems showed a moderate effect of reminder functions in reducing stress levels [79]. However, information on background, and tracking and reminder functions have been shown to improve long-term engagement within health apps [80], which might improve effectiveness through more intense and long-term usage. Similar to the results of Lau et al [34], most SMAs in this study were oriented toward self-help as merely 5 SMAs included the possibility to communicate synchronously (1/121, 0.8%) or asynchronously (4/121, 3.3%) with practitioners. This might be subject to change in future app development as a meta-analysis showed that professional guidance within mental health apps could significantly reduce stress levels compared with unguided apps (*g*=0.57 vs *g*=0.24) [79].

Support in terms of app communities was implemented in 16.5% (20/121) of the included SMAs. This is a positive trend compared with earlier findings showing only 4% of all included mindfulness-based apps providing this kind of support [78]. Since the availability of a community has beneficial effects on user engagement [80] and social support can positively influence the stress response [81], this seems to be a desirable trend.

Even though chronic stress is a major public health problem [1,9,12], only 3 SMAs were developed by institutions in the public sector (eg, universities or health authorities) and no SMA was officially certified (eg, according to the European Union Medical Device Regulation). This, together with the finding that only 5 SMAs were evaluated in randomized controlled trials, could indicate that thoroughly developed and evaluated apps might not find their way into the most popular app stores.

This study also focused on the declaration of the privacy and safety features within each identified SMA. The high percentage of SMAs providing a privacy policy (111/121, 91.7%) is promising. However, for 63.6% (77/121) of SMAs, no active confirmation of informed consent was required, and for 71.9% (87/121) of SMAs, there was no transparency regarding the right of withdrawal of informed consent. Data sharing with third parties was disclosed in the privacy policies of 17 (14.0%) SMAs. Regarding the actual practice of data security measures, Huckvale et al [40] showed that user data of health apps (depression and smoking cessation) have been shared with third parties, even without the necessary disclosure in the privacy policy. These results might be transferable to other health apps, including SMAs. Since the lack of data security is a common reason for user dissatisfaction with health apps and leads to the app being discontinued [82], improving data security measures may lead to increased engagement.

### Quality

The 121 included SMAs showed an acceptable to good overall quality (mean score 3.59, SD 0.50). The scores of the dimensions functionality and esthetics were above the cutoff value of 3.5. The scores of the dimensions engagement (mean 3.05, SD 0.78) and information (mean 3.42, SD 0.46) did not exceed this cutoff score. Overall quality was similar to that of other (mental) health apps, such as mindfulness apps (mean score 3.66, SD 0.48 [37]), physical activity apps (mean score 3.60, SD 0.59 [83]), depression apps (mean score 3.01, SD 0.56 [53]), or apps for posttraumatic stress disorder (mean score 3.36, SD 0.65 [84]). The rating below the cutoff score in the information dimension was also consistent with the findings of previous systematic reviews [33,34,37,83]. One explanation is the limited evidence base of SMAs. Only 9% of the included SMAs were scientifically evaluated. The rating below the cutoff score in the dimension engagement implies that the content and functions of SMAs might currently not be sufficient to bind the users in the long term. Implementation of diverse content or the possibility of personalization could help as these aspects are particularly relevant for the users of mental health apps [82].

### Theory-Based Stress Management Strategies

The examination of 3 types of theory-based stress management strategies resulted in 2.9 strategies per app. This is similar to the results in the study by Christmann et al [31], who reported 2.8 stress management strategies per app in their content analysis. Three of the four most implemented strategies are similar to the present results: meditation or mindfulness, breathing, and music (all categorized as regenerative stress management strategies). The results showed that instrumental and mental stress management strategies, which tend to be designed for prevention, are implemented less often than regenerative stress management strategies, which tend to be used for calming down after exposure to stress [13]. Therefore, the increased implementation of instrumental and mental strategies should be considered for a holistic approach to stress management in SMAs that seem to be relevant for effective prevention and coping with stress [13,15].

### Evidence Base and Long-Term Availability

For 11 of the 121 (9.1%) SMAs, a scientific evaluation could be found. Moreover, 5 (4.1%) of the SMAs were evaluated in randomized controlled trials and 1 (0.8%) in a partially randomized controlled trial showing improvement in different outcomes such as stress, self-efficacy, mindfulness, anxiety, and depressive symptoms [59-65]. Previous reviews of mental health apps for other target groups included similar or even fewer efficacy studies [37,53,83-86]. This might be explained by the high rate of updates and the high volatility of apps [34,44,45], which could also be confirmed in this study. Of 163 apps, 13 (8.0%) became unavailable during the app rating period, and only 62.0% (75/121) of all SMAs were still available after 2 years, with most of them (64/75, 85.3%) being updated. This poses a great challenge for not only users and health professionals, but also researchers regarding the use, recommendation, and evaluation of SMAs or other health apps [44]. In addition, the trustworthiness of the information about the content and functions within app descriptions is questionable. In this study, 163 apps were included based on the information in the description. However, 24 (14.7%) apps had to be excluded after downloading because the actual content did not meet the previously described content. This confirms the findings of Coulon et al [33], who found that 33% of SMAs did not contain the content advertised in their descriptions. Potential users must check any eligible app for accuracy after overcoming the hurdles of downloading the app, installing the app, and, if required, registering an account. New evaluation frameworks are needed and do exist, but in a systematic review, it was concluded that none out of 45 evaluation frameworks for medical apps was rated as being fully suitable [87]. A different approach to deal with the fast-moving nature of apps has been proposed by a group of international and diverse stakeholders [88]. They harmonized elements of different frameworks into 5 priority levels (background info, data privacy and security, app effectiveness, user experience and adherence, and data integration) with the aim to enable informed app decision-making rather than to constantly evaluate the apps. 

### Overview of the Implications of the 5 Top-Rated SMAs

By presenting SMAs with top-rated MARS-G quality together with their characteristics in a comparative overview, a broad information and decision basis can be provided for researchers, health professionals, and users. The 5 top-rated SMAs showed both common characteristics and consistencies with existing evidence. Three of the 11 evidence-based apps were rated in the top 5 SMAs in terms of quality. Furthermore, some strategies, functions, and technical aspects previously shown to be effective in reducing stress or shown to improve engagement were found among the top-rated apps, such as providing psychoeducation [80], including breathing [89] and mindfulness [64], providing monitoring and tracking [80], using reminders [79], tailoring the content to the users’ needs [90,91], and providing technical guidance and a privacy policy [82]. Across all SMAs and within the 5 top-rated SMAs, there were some aspects not covered by the MARS-G (eg, theory-based stress management strategies such as guided imagination, visualization, or music). This demonstrates the value of the overview of the top-rated SMAs (eg, compared with simple app rankings), in particular when special weight is given to certain aspects or app characteristics. In addition, the joint presentation of MARS-G content and additional uncovered aspects reveals certain revision potentials of the MARS-G.

### Limitations

There were some limitations. First, owing to the rapid development of the app market and the short lifespan of apps, the content and quality of the reviewed SMAs may have already changed, some SMAs may no longer be available, or new SMAs may have been launched. However, this seems to be a challenge in general for health technology evaluation [44,87], and a screenshot of the current status might help to derive implications for improving SMA quality and effectiveness, and improving the evaluation frameworks for apps in the future. Second, the search results per search term were limited to 200 results and screening was based on the titles and descriptions of the apps. It is possible that apps that met the inclusion criteria were overlooked because relevant information was not provided or the word “stress” was not present in the titles or descriptions. Furthermore, only SMAs that were free of cost or provided a free basic version were evaluated. Further evaluation of paid (full version) SMAs could show whether there is a difference in quality, declaration of data privacy and security features, or access to professional support. Third, there was only a descriptive evaluation (not a technical evaluation; eg, for data privacy issues), and no conclusions about the overall effectiveness of the included SMAs could be drawn. Fourth, the number of SMAs including “breathing” as a strategy differed in the MARS rating and in the assessment of theory-based stress management strategies. Therefore, it should be emphasized that the discriminant differentiation of the related and partially overlapping concepts or strategies of “mindfulness” and “breathing” cannot be assessed conclusively (especially considering that “breathing” can also be practiced as a concrete strategy within the context of mindfulness). However, the fact that “breathing” and “mindfulness” were listed in the top 3 strategies remains unchanged. Finally, the review and evaluation of each app took an average of 30 minutes. It is possible that specific content could not be discovered owing to the limited amount of time spent evaluating each app.

### Conclusion

In this comprehensive review including a systematic search and a standardized multidimensional assessment, the overall quality of 121 SMAs was rated as acceptable to good, with a rating below the cutoff score in the dimensions of information quality and engagement. The top-rated apps included psychoeducation, breathing and mindfulness, monitoring, reminder functions, tailoring, technical guidance, and a privacy policy. However, even though most SMAs provided a privacy policy, there is still a need for better personal data protection and transparency of data processing, such as the use of a password or information about data sharing with third parties. Theory-based strategies were mostly regenerative stress management strategies. For a holistic stress management approach, SMAs could benefit from the integration of more mental and instrumental stress management strategies. The evidence base for 11 (9.1%) of the 121 included apps showed that SMAs can reduce stress and improve further outcome variables such as self-efficacy, mindfulness, anxiety, and depressive symptoms. Moreover, SMAs have high scalability. Therefore, they have a high potential to reach and help a broad audience coping with increasing stress and demands in their work and daily living. However, the rather moderate information quality, the scarce evidence base of the included SMAs, and the fact that many SMAs changed or were unavailable after a 2-year period pose challenges for users and health professionals who are searching for high-quality apps that are effective and for long-term use. The common characteristics of SMAs with top-rated quality and evidence base of SMAs can be used as guidance for this search or even for SMA development. In addition, it is difficult for researchers to keep up to date with the latest research in this volatile field and provide potential users with helpful information. Enhanced evaluation frameworks are needed that might complement or even advance the idea of a continuous effectiveness and quality assessment to an approach that enables informed decision-making.

## Appendix

Multimedia Appendix 1 Frequency of declared data privacy and security features based on the German Mobile Application Rating Scale presented as a bar graph. The asterisk (*) indicates additional features that have been added to the original list.

Multimedia Appendix 2 Frequency of the implemented strategies according to the German Mobile Application Rating Scale presented as a bar graph.

Multimedia Appendix 3 Quality ratings of all included stress management apps.

Multimedia Appendix 4 Theory-based stress management strategies according to Kaluza [13] and Christmann et al [31] presented as a bar graph. Regenerative stress management strategies (RSMSs) are indicated with black, mental stress management strategies (MSMSs) are indicated with vertical lines, and instrumental stress management strategies (ISMSs) are indicated with horizontal lines. The asterisk (*) indicates theory-based stress management strategies that are also included in the German Mobile Application Rating Scale.

Multimedia Appendix 5 Overview of stress management apps with an evidence base.

## Abbreviations

ICC: interclass correlation
MARS: Mobile Application Rating Scale
MARS-G: German Mobile Application Rating Scale
SMA: stress management app

## Data Availability

Data will be made available to researchers who provide a methodologically sound proposal, not already covered by other researchers. All requests should be directed to the corresponding author. Data requestors will need to sign a data access agreement. Provision of data is subject to data security regulations. Support depends on available resources.
