# Supplementary figures and images for: Stress Management Apps: Systematic Search and Multidimensional Assessment of Quality and Characteristics
Source: JMIR Mhealth Uhealth. 2023 Aug 29;11:e42415. doi: 10.2196/42415 (PMC10498318; doi:10.2196/42415)

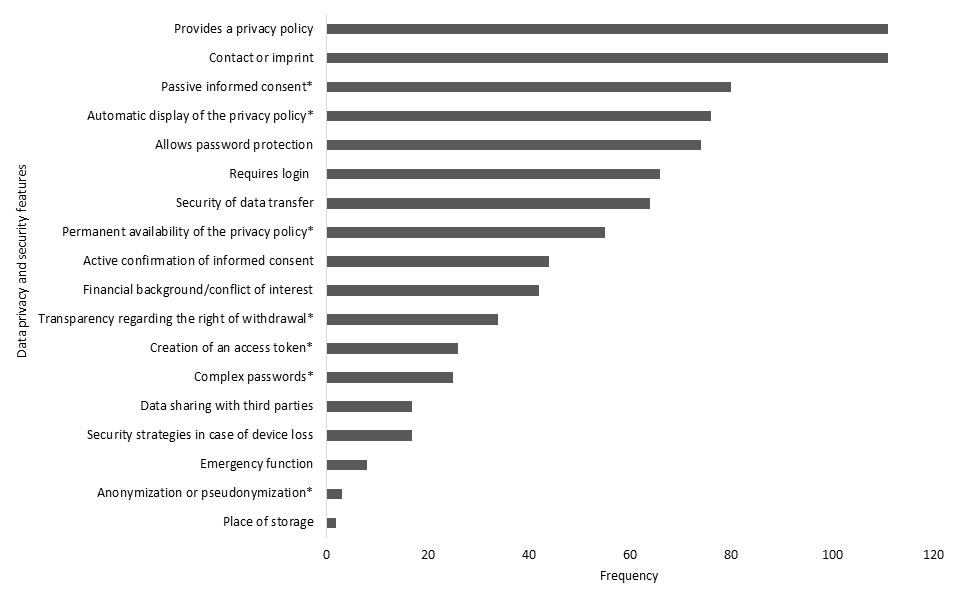

Supplement: Multimedia Appendix 1 [file mhealth_v11i1e42415_app1.png]

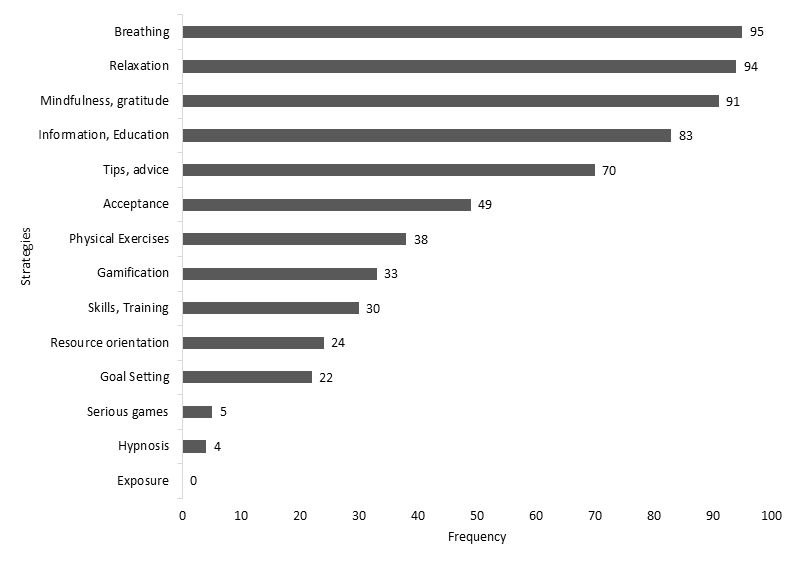

Supplement: Multimedia Appendix 2 [file mhealth_v11i1e42415_app2.png]

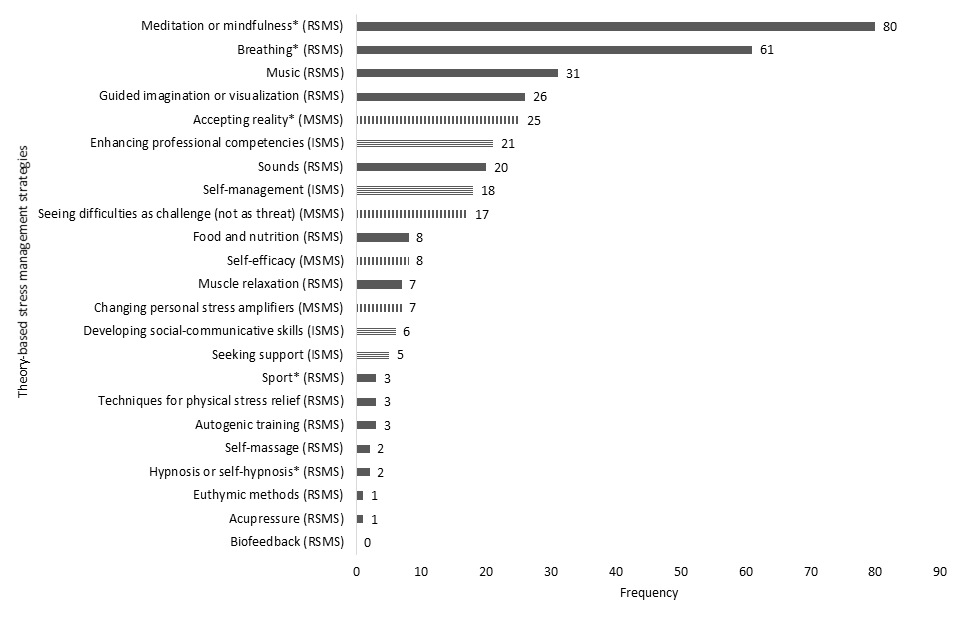

Supplement: Multimedia Appendix 4 [file mhealth_v11i1e42415_app4.png]
