# Supplementary material for: Stress Management Apps: Systematic Search and Multidimensional Assessment of Quality and Characteristics
Source: JMIR Mhealth Uhealth. 2023 Aug 29;11:e42415. doi: 10.2196/42415 (PMC10498318; doi:10.2196/42415)
Supplement: Multimedia Appendix 3 [file mhealth_v11i1e42415_app3.pdf]

Multimedia Appendix 3. Quality ratings of all included stress management apps.

App name	Overall quality	User Engagement	Functionality	Aesthetics	Information quality
Happify: bei Ärger und Stress	4,36	4,30	4,63	4,50	4,00
Sanvello: Stress & Anxiety Help	4,22	4,10	4,38	4,83	3,57
Headspace: Meditation & Schlaf	4,21	3,80	4,50	4,83	3,71
go4health – gesund leben	4,19	4,00	4,75	4,17	3,86
BamBu Meditation & Achtsamkeit	4,16	4,30	4,25	4,50	3,57
Ease: Achtsamkeit & Meditation	4,13	3,90	4,88	4,33	3,43
Aurum - Stress, Anxiety, Mental Health, Therapy.	4,12	3,60	4,75	4,50	3,64
Pocketcoach - Anxiety Helper	4,12	4,00	4,63	4,50	3,36
Balloon – Meditation für alle	4,10	3,80	4,88	4,00	3,71
Keep Yoga - Yoga & Meditation	4,09	4,60	4,13	4,50	3,14
DeStressify-FREE Stress Relief	4,08	4,10	4,38	4,00	3,86
Sanvello (formerly Pacifica)	4,08	4,00	4,38	4,17	3,79
Mindshine - Self Care Coaching	4,07	4,10	4,50	4,67	3,00
Meditopia: Meditation Deutsch	4,06	3,90	4,63	4,50	3,21
Mind Ease: Anxiety Relief	4,06	4,20	4,25	4,50	3,29
Wellness Coach:meditation.live	4,06	3,70	4,50	4,67	3,36
Headspace: Meditation & Schlaf	4,05	4,00	4,50	5,00	2,71
reviti life	4,02	4,30	4,38	4,33	3,07
Balance: Meditation	3,99	4,10	4,75	4,67	2,43
Meditopia: Meditation Deutsch	3,96	3,90	4,63	4,33	3,00
BamBu: Meditation für Entspannung & Schlaf	3,94	4,00	4,50	4,33	2,93

Mindshine - Personal Training for the Mind	3,93	3,90	4,00	4,67	3,14
Wellness Coach: Meditation.live	3,92	3,70	4,38	4,33	3,29
DeStressify Stress Relief	3,88	3,40	4,00	3,83	4,29
Meditation & Klänge von Verv	3,87	3,20	4,50	4,83	2,93
Aura: Sleep & Mindfulness	3,81	4,20	4,13	4,50	2,43
Meditation, Sleep & Focus App	3,81	3,20	4,50	4,67	2,86
Pocketcoach - Anxiety Helper	3,80	3,50	4,38	4,17	3,14
Breethe - Calm Meditation, Sleep & Mindfulness	3,78	3,70	4,00	4,83	2,57
Stop, Breathe & Think: Meditation & Mindfulness	3,77	3,50	3,88	4,00	3,71
Insight Timer - Meditation	3,75	3,70	4,50	4,17	2,64
Stress-Mentor	3,75	3,70	3,88	4,00	3,43
Happify: bei Ärger und Stress	3,74	4,10	4,38	4,33	2,14
Wim Hof Method	3,72	2,50	4,38	4,17	3,86
AOK Relax	3,72	3,30	4,75	3,83	3,00
Woebot: Your Self-Care Expert	3,72	3,30	4,25	3,83	3,50
Ten Percent Happier - Meditation & Sleep	3,71	3,30	4,75	4,17	2,64
Mindrise: Sleep, Meditation, Music & Astrology	3,70	3,80	4,13	4,50	2,36
Emoski	3,69	4,10	3,88	4,00	2,79
Shine: Calm Anxiety & Stress	3,68	3,60	4,25	4,17	2,71
NeuroNation Ease: Achtsamkeit & Meditation	3,68	3,40	4,50	4,17	2,64
Peace: Calm, Sleep, Meditation	3,67	3,60	3,88	4,50	2,71
AOK Lebe Balance	3,66	3,90	3,88	4,00	2,86
MYditation - Meditation für Entspannung & Schlaf	3,65	3,70	3,75	4,17	3,00
Meditation und Entspannung mit Cosmiq	3,64	3,10	4,25	4,33	2,86

Insight Timer - Free Meditation App	3,63	3,70	4,25	4,00	2,57
De-StressMe: CBT Tools to Manage Stress	3,61	4,00	4,50	4,00	1,93
eleMental	3,60	3,80	3,50	4,33	2,79
Meditation, Sleep & Relax - Urban Yogi mindfulness	3,60	3,90	3,88	4,00	2,64
Balloon - Meditation für alle	3,60	3,40	4,38	4,33	2,29
Breethe: Meditation & Schlaf	3,55	3,70	4,25	4,17	2,07
7Mind: Meditation reinvented	3,51	3,40	3,88	4,33	2,43
MoodSpace - Stress, anxiety, & low mood self-help	3,49	3,30	3,38	4,00	3,29
Youper - Emotional Health	3,47	3,20	3,75	4,00	2,93
Resility Personal Biofeedback	3,47	2,40	4,50	4,33	2,64
Serenity: Guided Meditation & Mindfulness	3,47	3,10	4,25	3,67	2,86
Music Zen: Entspannende Klänge	3,42	3,00	4,38	4,50	1,79
Present Mind - Mindfulness	3,41	2,70	4,38	3,33	3,21
Welzen: meditations to relax, focus & sleep better	3,40	3,30	4,13	3,83	2,36
Key Meditation	3,40	2,50	4,38	4,00	2,71
Rize: Relaxation Mental Stress	3,40	3,50	3,88	3,50	2,71
WhyFit	3,39	2,70	4,13	3,67	3,07
Breathe+	3,37	3,40	4,88	3,50	1,71
Calm in the Storm: Stress Management and Relaxation	3,37	3,10	3,50	3,67	3,21
StressLess	3,36	2,60	4,00	3,50	3,36
Free Guided Meditation and Relaxation	3,36	3,20	4,38	4,17	1,71
AOK Relax	3,34	2,60	4,63	3,00	3,14
7Mind Meditation & Achtsamkeit	3,29	3,00	4,25	4,00	1,93
DayStress Relief	3,29	2,90	4,75	3,50	2,00

Relax Lite: Stress and Anxiety Relief	3,26	2,70	4,50	4,00	1,86
Serenity: Meditation deutsch	3,26	2,80	4,63	3,67	1,93
Guided Meditation & Relaxation	3,24	2,70	4,75	4,00	1,50
7pranayama - Yoga Daily Breath Fitness Yoga & Calm	3,23	3,20	3,88	2,83	3,00
Resility Stress Management & Biofeedback	3,22	3,10	4,13	3,50	2,14
Meditation for Anxiety and Stress	3,20	2,80	4,00	3,00	3,00
Medativo - Meditation Timer	3,20	3,10	4,13	3,50	2,07
RelaxApp - Meditation Relaxation Chromotherapy	3,19	3,00	4,38	3,33	2,07
Restful	3,18	3,00	3,50	4,17	2,07
Wildfulness 2 - Natur klingt	3,16	1,70	4,50	4,50	1,93
Mindfulness & Guided Sleep Meditation	3,16	2,60	3,50	3,67	2,86
Relaxation Coach	3,15	2,10	4,38	4,00	2,14
The SoSial	3,15	2,30	3,63	3,83	2,86
Wellbody	3,14	2,40	4,25	4,00	1,93
Prime - Psychological Security	3,13	2,80	3,75	3,33	2,64
Calmcast	3,11	2,80	4,25	3,33	2,07
BrainTap Pro	3,11	2,40	4,38	3,67	2,00
Mindfulness Meditation & Exercises	3,10	2,40	4,13	3,67	2,21
Happy Being- Meditate, De-stress, Sleep, Wellbeing	3,08	3,30	3,50	3,67	1,86
Life Coach, CBT, Emotional Therapy by Libby Seery	3,08	2,40	4,38	3,33	2,21
MoodMission - Cope with Stress, Moods & Anxiety	3,08	2,30	3,13	3,67	3,21
Stress Less Cards	3,04	2,40	4,63	3,83	1,29
Fantasiereise mit Aut Training	3,03	2,10	4,38	3,00	2,64

Meditation, Mindfulness & Relaxation by MT	3,01	2,70	3,88	3,17	2,29
Headspax - Meditation , Mindfulness & Motivation	3,00	1,80	5,00	3,00	2,21
HeartsApp	2,97	2,50	3,88	3,50	2,00
Pocket Meditation - Daily Stress & Anxiety Relief	2,96	2,00	4,50	4,33	1,00
Otemi VR	2,95	2,20	4,00	3,67	1,93
MindPilot - Mindfulness App	2,94	2,80	3,88	2,67	2,43
Stress Less Cards	2,94	2,20	4,38	3,17	2,00
Mindset	2,93	3,10	3,38	3,33	1,93
Otemi	2,92	2,10	4,00	3,67	1,93
wingwave	2,92	2,00	3,88	3,17	2,64
Guided Meditation Free App	2,90	2,30	4,25	3,33	1,71
Meditation in Motion	2,85	2,30	3,50	3,17	2,43
Meditieren lernen	2,82	1,60	4,38	2,50	2,79
Atemübungen und Pranayama Techniken	2,78	2,50	4,25	2,67	1,71
Zen	2,75	2,10	3,63	4,00	1,29
Calmcast	2,74	2,70	3,50	2,83	1,93
Entspannung PUR	2,67	2,50	4,00	2,33	1,86
Stressbusters Wellness	2,65	2,60	3,13	2,50	2,36
Ease My Stress & Anxiety	2,64	1,40	4,00	3,17	2,00
Mindfulness2Go	2,63	2,10	3,25	2,67	2,50
My Healthy Treasure Chest	2,59	1,80	4,13	2,00	2,43
Mindful meditate, relax, sleep	2,56	1,90	4,00	2,83	1,50
Free relaxation techniques.	2,38	1,70	4,00	2,67	1,14
Relaxation techniques	2,35	1,50	3,50	2,33	2,07
Relaxation Techniques & Stress Management- Effective Techniques & Tips	2,26	2,30	2,88	1,67	2,21
Mindfulness2Go	2,19	1,50	2,63	2,33	2,29
Entspannungstechniken	2,17	1,50	3,88	2,00	1,29

Stress Relief Hypnosis: Anxiety, Relax & Sleep	2,07	1,70	3,13	2,17	1,29
ShrinkinaBox	2,06	2,40	2,63	1,50	1,71
