# Supplementary material for: Stress Management Apps: Systematic Search and Multidimensional Assessment of Quality and Characteristics
Source: JMIR Mhealth Uhealth. 2023 Aug 29;11:e42415. doi: 10.2196/42415 (PMC10498318; doi:10.2196/42415)
Supplement: Multimedia Appendix 5 [file mhealth_v11i1e42415_app5.pdf]

Multimedia Appendix 5. Overview of stress management apps with an evidence base.

Reference	Study type/ design	App	App usage (week)	Sample	Mean age (SD)	Female (%)	Target group	Measure-ment time points	Measured variables	Main results
Bakker & Rickard, 2019 [65]	Panel study	MoodMission	4 (30 days)	671	27 (10.9)	71	General population	Baseline<br><br>Post intervention	-depression<br>-anxiety<br>-wellbeing	*Only within group comparisons:*<br>Significant differences only for wellbeing in favor of the app
Flett et al., 2019 [66]	Randomized controlled trial	Head-space[1]	1.5 (10 days)<br><br>+30 days app availability	145<br>IG (App): 72<br><br>CG (Organization app): 73	20 (2.01)	70	University students	Baseline<br><br>Post intervention (after 10 days)<br><br>Follow up (after 40 days)	-depressive symptoms<br>-anxiety<br>-stress<br>-college adjustment<br>-flourishing<br>-resilience<br>-mindfulness	*Between group comparisons:*<br>Significant differences in depressive symptoms, anxiety, stress,<br>college adjustment and mindfulness in favor of the app at post intervention<br><br>Significant differences in anxiety, stress,<br>college adjustment, resilience and mindfulness in favor of the app at follow up
Fuller-Tyszkiewicz et al., 2020 [63]	Randomized controlled trial	StressLess	5	183<br>IG (App): 73<br><br>CG (App for self-monitoring): 110	40 (6.27)	95	Caregiver for family/ friends with a physical/ mental disability	Baseline<br><br>Post intervention<br><br>Follow up (3 months post intervention)	-depression<br>-anxiety<br>-stress<br>-well-being<br>-self-esteem<br>-optimism<br>-primary and secondary control<br>-social support	*Between group comparisons (baseline – post intervention):*<br>Significant differences in depression, anxiety, stress and well-being in favor of the app and secondary control in favor of the CG<br><br>*Between group comparisons (post intervention – follow up):*<br>Significant differences in depression, well-being, self-esteem, optimism and social support in favor of the app
Moberg et al., 2019 [62]	Randomized controlled trial	Sanvello (former name: Pacifica)	4	500<br>IG (App): 253<br><br>WCG: 247	30 (10.9)	75	Adults with mild to moderate anxiety or depression	Baseline<br><br>Post intervention<br><br>Follow-up (2 months post intervention; but only IG)	-stress<br>-anxiety<br>-depression<br>-self-efficacy	*Between group comparisons:*<br>Significant differences for all variables in favor of the app at post intervention
Möltner et al., 2018 [61]	Randomized controlled trial	7mind	2	306<br>IG (App): 146<br><br>WCG: 160	43 (10.36)	69	Employed individuals	Baseline<br><br>Post intervention	-Mindfullness<br>-Work engagement<br>-Job satisfaction<br>-Innovation and creativity<br>-Emotional exhaustion<br>-Emotional intelligence<br>-Self efficacy	*Between group comparisons:*<br>Significant differences for all variables in favor of the app
Nagel et al., 2019 [60]	Partially randomized trial	AOK Relax	6	94<br>IG 1 (only app): 17<br><br>IG 2	 21 (2.46)<br><br>21 (2.00)<br><br>22 (2.73)	 100<br><br>100<br><br>100	University students	Baseline<br><br>Post intervention	Perceived stress<br>Mindfulness<br>Well being	*Only within group comparisons:*<br>- Significant differences only in IG 3 for all variables in favor of the program

				(App + SMP): 8<br><br>IG 3 (only SMP): 12<br><br>CG: 57	22 (2.63)	63				
Parks et al., 2018 [59]	Randomized controlled trial	Happify	8	4,485 IG (Platform + App): 2,275<br><br>CG (psychoeducation): 2,210	NA	86	General population	Baseline<br><br>Post intervention	-depressive symptoms<br>-anxiety symptoms<br>-resilience (including items on stress, positive emotionality, optimism)	*Between group comparisons:* Significant differences for all variables in favor of the app

SD, standard deviation; IG, intervention group; CG, control group; WCG, waitlist control group; NA, not available.

Note: The remaining four of the 11 scientific publications are not listed here in detail, as they are pilot studies [67-69] and a conference paper [70].

[1] This study was chosen exemplarily. The Headspace app has been evaluated in several other studies [64,71-77]. This table may be incomplete as no systematic search was carried out.
